# ﻿A taxonomic study of *Gandaritis flavomacularia* and related species (Lepidoptera, Geometridae), with description of a new species from western China

**DOI:** 10.3897/zookeys.1255.165076

**Published:** 2025-10-15

**Authors:** Boxin Wen, Rui Cheng

**Affiliations:** 1 State Key Laboratory of Animal Biodiversity Conservation and Integrated Pest Management, Institute of Zoology, Chinese Academy of Sciences, No.1, Beichen West Road, Chaoyang District, Beijing, 100101, China Institute of Zoology, Chinese Academy of Sciences Beijing China; 2 Institute of Xizang Plateau Ecology, Xizang Agricultural & Animal Husbandry University, Linzhi, Xizang, 860000, China Xizang Agricultural & Animal Husbandry University Linzhi China

**Keywords:** COI, DNA barcoding, Larentiinae, morphology, systematics, taxonomy

## Abstract

The taxonomic status of *Gandaritis flavomacularia* has recently undergone revision. This study evaluates the validity of *G. flavomacularia* based on both morphological and molecular evidence. Furthermore, a new, closely related species, *Gandaritis stueningi* Wen & Cheng, **sp. nov.** from Sichuan, China, is described, supported by both molecular and morphological data. Key morphological characters, including the male and female genitalia, are illustrated and compared with those of three related species.

## ﻿Introduction

**Figure 1.**
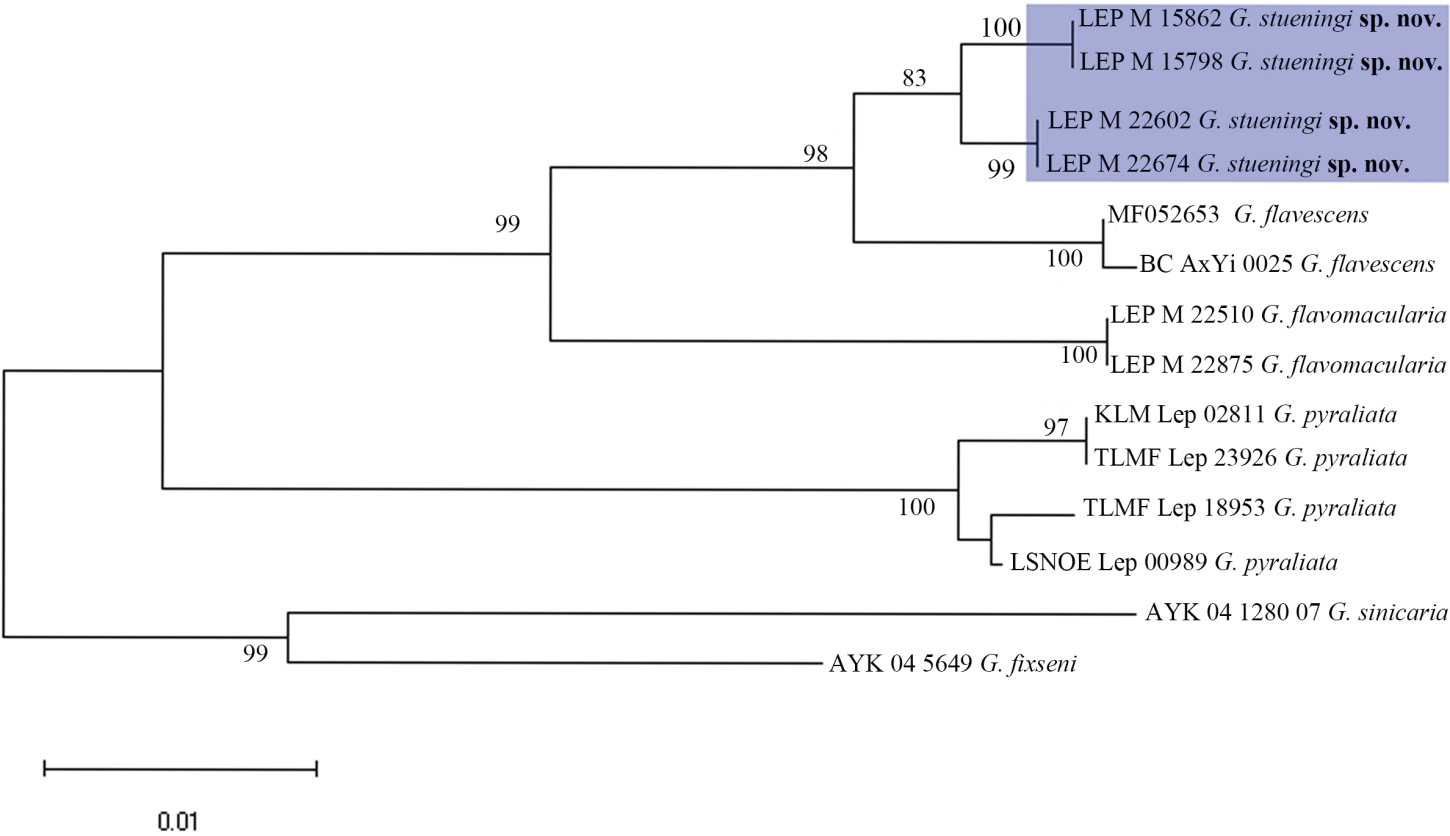
Neighbour-joining (NJ) tree of selected *Gandaritis* based on the Kimura two-parameter model.

*Gandaritis flavomacularia* Leech, 1897 (Larentiinae) was originally described on the basis of two type specimens (a male and a female) from Wa-Shan, Sichuan, western China. Prout (1915) later transferred this species to the genus *Lygris* Hübner, 1825, citing the ‘absence of secondary sexual characters’. Herbulot (1964) synonymized *Lygris* with *Eulithis* Hübner, 1821, resulting in the new combination: *Eulithis flavomacularia*. This taxonomic treatment was followed by Parsons et al. (1999) and Rajaei et al. (2022). Xue and Zhu (1999) subsequently reassigned *flavomacularia* to *Gandaritis* based on a comprehensive suite of characters, including wing venation and genital morphology. Molecular data further support the validity of *G. flavomacularia* within *Gandaritis* (Fig. 1). This species has two close relatives: *G. tristis* (Prout, 1938) and *G. flavescens* Xue, 1992. *Gandaritis tristis* is known from the nearby holotype locality (Ta-tsien-lu, Sichuan, China), shares a similar wing pattern and also lacks secondary sexual characters, but exhibits a distinctive yellow costal patch extending to the wing apex. *Gandaritis flavescens* is known from Sangzhi, Hunan, China, and shares strongly biangulate hindwing discocellulars with *G. flavomacularia*.

Stüning and Fu (2019) affirmed the findings of Xue and Zhu (1999) and provided a comprehensive review of the genus’s taxonomic history, establishing a foundation for further research. Within *Gandaritis*, the presence or absence of secondary sexual characters and the shape of the hindwing discocellulars exhibit overlapping variation (Xue and Zhu 1999), indicating that reliance solely on the ‘absence of secondary sexual characters’ may be misleading. For instance, both *G. tristis* and *G. fixseni* (Bremer, 1864) lack secondary sexual characters, while strongly biangulate hindwing discocellulars—characteristic of *G. flavomacularia*—also occur in *G. tricedista* (Prout, 1938), *G. flavescens*, *G. nigroagnes* Xue, 1999, and *G. agnes* (Butler, 1878).

Currently, *Gandaritis* comprises 22 species (Choi 2001; Stüning and Fu 2019; Rajaei et al. 2022), 16 of which occur in China. Typical diagnostic characters of *Gandaritis* include: the central fascia on the forewing, comprising dots or a band, is strongly projected outwardly on the middle of veins M_2_ or M_3_; termen of both fore- and hindwings usually shows an orange band with blackish dots; the male genitalia are similar to those of *Eulithis*, but distinguished by the long anellus lobe that exceeds half the length of the tegumen, the broad apical part of the anellus lobe with long, hair-like setae, the costa of the valva is slightly expanded medially, and the vesica lacks cornuti (Prout 1915; Choi 2001).

During further examination of *Gandaritis* specimens from Institute of Zoology, Chinese Academy of Sciences, Beijing, China (IZCAS) and Zoologisches Forschungsmuseum Alexander Koenig, Bonn, Germany (ZFMK), we discovered an undescribed species morphologically similar to *G. flavomacularia* and *G. tristis*, sympatric with them in western China. Here, we describe this new species, provide comparative illustrations of external and genital morphology for both sexes, and present DNA barcode evidence supporting its distinct status.

## ﻿Material and methods

All studied specimens, including the types of the new species, are deposited in IZCAS or ZFMK. Wing venation terminology follows the Comstock-Needham system (Comstock 1918), as adopted for Geometridae by Scoble (1992) and Hausmann (2001), while genitalia terminology follows Pierce (1914, reprinted 1976), Klots (1970), and Nichols (1989). Moths photographs were taken with a digital camera (Canon Pc1057), 20 cm LED Photography Shadowless Light (Color temperature: 6000–6500 K, 600 lm), and composite images were generated using Auto-Montage software (ver. 5.03.0061, Synoptics Ltd). Plates were compiled in Adobe Photoshop (ver. 2020).

A total of six DNA barcodes from IZCAS specimens were included in this study, comprising four sequences of the new species and two sequences of *G. flavomacularia*. A further eight sequences from four other *Gandaritis* species were obtained from the BOLD system (https://boldsystems.org/). Details of all specimens are summarized in Appendix 1. DNA extraction and sequencing followed the protocols described by Cheng et al. (2025). Sequences were aligned using MEGA 6.0 (Tamura et al. 2013) and a neighbour-joining (NJ) tree (Saitou and Nei 1987) was constructed based on Kimura two-parameter (K2P) distances (Kimura 1980).

## ﻿Systematics

### ﻿ Gandaritis stueningi

Taxon classificationAnimaliaLepidopteraGeometridae

Wen & Cheng sp. nov.

06AD5815-BE32-5178-AF81-5ED7F26BC016

https://zoobank.org/6DDE2CCF-828F-4A0E-AAA2-AA7FFA2D3DB9

Figs 2–6, 17–20, 25 Chinese common name: 斯氏枯叶尺蛾

#### Type material.

***Holotype***, ♂, **China**: **Sichuan** (IZCAS): • Pingwu, Wanglang, 2446 m, 21–22.VII.2016, leg. Cui Le, slide no. Geom-07564, IOZ LEP M 22602. ***Paratypes*: Shaanxi** (IZCAS): • 1 ♂, 1 ♀, Tiantaishan, Jialingjiang, 1062 m, 8–9.VIII.2014, leg. Xue Dayong, slide no. Geom-07566, IOZ LEP M 15798, 15862; • 1 ♀, Ningshan, Huoditang, 1550 m, 8.VII.2008, leg. Cui Junzhi; • 1 ♂, 1 ♀, (IZCAS, ex. ZFMK), Tapaishan im Tsinling Sued-Shensi, ca. 1700 m, 8.VIII.1936, leg. H. Höne, slide no. Geom-01949. **Shaanxi** (ZFMK), 3 ♀, Tapaishan im Tsinling Sued-Shensi, ca. 1700 m, 22–26.VII.1935, leg. H. Höne; • 4 ♂, 2 ♀, ibidem, 6–19.VIII.1935; • 6 ♂, 8 ♀, ibidem, 5–19.VIII.1936; • 1 ♀, Tapaishan im Tsinling Sued-Shensi, ca. 3000 m, 25.VII.1935, leg. H. Höne; • 3 ♂, 3 ♀, ibidem, 11–25.VIII.1936. **Gansu** (IZCAS), 8 ♀, Dangchang, Guanegou, 2045 m, 1–3.VIII.2016, leg. Cheng Rui & Jiang Shan; • 1 ♀, Wenxian, Liziba, 1971 m, 22–24.VIII.2014, leg. Li Xinxin; • 2 ♀, Wenxian, Qiujiaba, 2200 m, 16–19.VII.2003, leg. Wang Hongjian; • 1 ♀, Kangxian, Qinghe, 1400 m, 7.VII.1999, leg. Zhu Chaodong. **Sichuan** (IZCAS), 1 ♀, Pingwu, Wanglang, 2504 m, 23.VII.2016, leg. Cui Le, slide no. Geom-07563, IOZ LEP M 22674; • 10 ♀, Pingwu, Wanglang, 2410 m, 5.VIII.1999, leg. Zhou Xin.

#### Description.

***Head*.** Antennae filiform in both sexes, lacking cilia. Head coloration dark grayish-brown to blackish-brown. Labial palpus moderately long, extending beyond frons. Vertex grey-brown, intermixed with grayish-white scales.

***Thorax*.** Dorsally dark grayish-brown to blackish-brown. Leg coxae grayish-white, femora to tarsi pale yellowish-gray, intermixed with dark grayish-brown. Patagia sparsely mixed with yellowish scales. Forewing length: ♂ 26–27 mm, ♀ 29–31 mm. Wings broad; forewing with apex pointed, slightly protruding; outer margin gently curved. Hindwing termen arched. Discocellular of hind wing strongly biangulate; vein D_2_ distinctly longer than D_1_ and D_3_; base of M_2_ slightly close to M_3_. Wing omit comma color brown to blackish-brown. Forewing with white antemedial, medial, and postmedial lines. Antemedial line oblique outward from costa to discal cell, slightly thickened, bending inward within cell, slightly wavy to inner margin. Median line thickened at costa, forming an outwardly oblique wedge-shaped white patch, angled backward within discal cell and slightly wavy to inner margin. Postmedial line narrow white streak, tapering, extending obliquely to mid-vein M_2_, following part inclined to basal 1/3 of CuA_2_ or often invisible, then slightly thicken and wavy to inner margin. Submarginal and terminal lines absent. Fringes thickened with wing. ***Hindwing*.** Costa grayish-white from base to postmedial line; area below often tinged with yellow, sometimes expanding downward forming diffuse yellow patch at mid-wing. Discal spot round, dark brown, faint. Postmedial line stout, yellow, deeply curved, lower half slightly wavy. Submarginal line curved, composed of a series of crescent-shaped yellow spots. Costa yellow from submarginal line to apex. Fringes yellow at apex, remainder concolorous with wing. ***Forewing underside*.** Mostly yellowish; costa to discal cell intermixed with grayish-white and dark brown; distal part blackish. Dark brown patch present at middle of anterior margin of discal cell. Discal spot rounded, dark brown. Postmedial line blackish, anterior part very broad, forming a “>”-shaped angle; posterior part tapering and bent outward near inner margin. Submarginal line blackish, curved, discontinuous on veins, almost merged with blackish distal part near costa. Underside of male forewing without secondary sexual characteristics. ***Hindwing underside*.** Grayish-white to pale grayish-yellow, mixed with brown. Postmedial and submarginal lines blackish; the former deeply curved, with posterior part broader; the latter broad and arcuate, interrupted on veins. Discal spot more distinct than upperside. ***Abdomen*.** Its dorsal side dark grayish-brown to blackish-brown, intermixed with grayish-white scales; ventral sides grayish-white. Setal patch on male third sternite absent.

**Figures 2–16.**
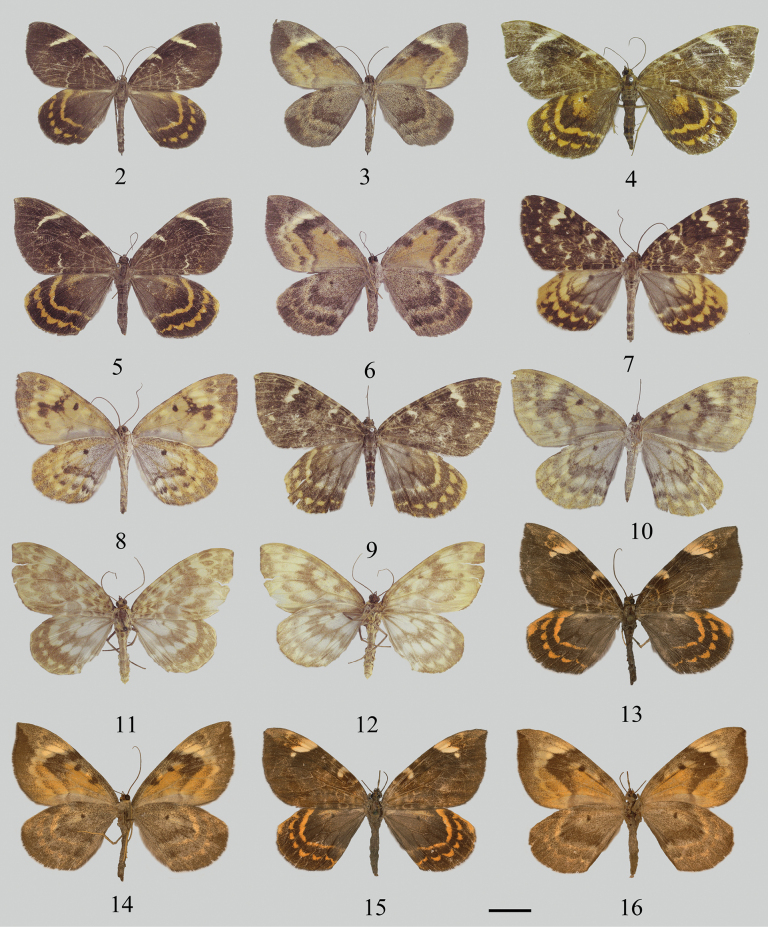
Wing pattern of *Gandaritis* species. 2–6 *G. stueningi* sp. nov. 2. Holotype, male, upperside (in: IZCAS); 3. Ibidem, underside; 4, 5. Paratype, female, upperside (in: IZCAS); 6. Ibidem, underside; 7–10. *G. flavomacularia*. 7. Male, upperside (in: IZCAS); 8. Ibidem, underside; 9. Female, upperside; 10. Ibidem, underside; 11, 12. *G. flavescens*. 11. Holotype, male, upperside (in: IZCAS); 12. Ibidem, underside; 13–16. *G. tristis*. 13. Male, upperside (in: ZFMK); 14. Ibidem, underside; 15. Female, upperside (in: ZFMK); 16. Ibidem, underside. Scale bars: 1 cm.

**Figures 17–26.**
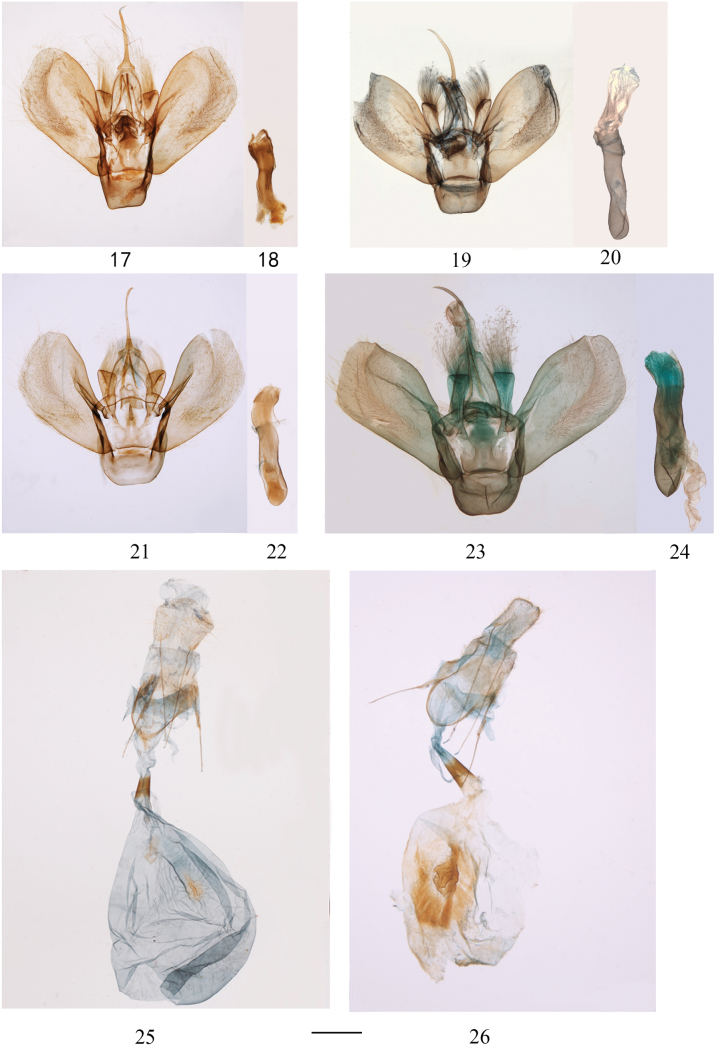
Male and female genitalia. 17–24. Male genitalia. 17–20. *G. stueningi* sp. nov.; 17, 18. Holotype (slide no.: 07564); 19, 20. Paratype (slide no.: 01949); 21, 22 *G. flavomacularia* (slide no.: 07562); 23, 24. *G. flavescens* (slide no.: 02073); 25–26. Female genitalia. 25. *G. stueningi* sp. nov., paratype (slide no.: 07563); 26. *G. flavomacularia* Leech (slide no.: 07561). Scale bars: 0.5 mm.

***Male genitalia*** (Figs 17–20). Uncus very long, narrow, apex pointed. Tegumen simple. Juxta broad, bearing well sclerotized conical process medially on posterior margin. Anellus lobe developed, extremely robust, truncate at apex, densely covered with long hairs, tip swollen. Saccus short and broad. Valva wide and simple. Aedeagus stout, large, its middle part broad; vesica large, membranous, lacking cornuti. Manica with a short, tongue-like process, surrounding membrane finely spined.

***Female genitalia*** (Fig. 25). Papilla anales simple. Apophyses posteriores and anteriores slender and elongate. Lamella antevaginalis narrow, semicircular, sclerotized band. Ductus bursae approximately two-thirds length of elliptical corpus bursae; colliculum present at lower half. Corpus bursae bears an irregularly shaped signum, located at middle area and ventral side, composed of numerous spinules.

#### Diagnosis.

This species is morphological similar to *G. flavomacularia* (Figs 7–10). Compared to *G. flavomacularia*, the wing color of *G. stueningi* sp. nov. is darker; the white streak along the forewing postmedial line is narrower, straighter, and more steeply inclined. In *G. stueningi* sp. nov., the submarginal and terminal lines are absent, whereas in *G. flavomacularia*, the submarginal line consists of a series of white spots of various sizes, and the terminal line forms a series of yellow dots at the end of veins. On the underside, *G. stueningi* sp. nov. is darker, and the postmedial line is more strongly protruding, forming a “>” shape, while in *G. flavomacularia*, the postmedial line is less convex, and connected to the submarginal line by a black-brown spot. The genitalia also differ: the male saccus in the new species is narrower than that in *G. flavomacularia* (Figs 21, 22) and in the female of the new species, the antrum is shorter and the signum more slender than in *G. flavomacularia* (Fig. 26). Unlike *G. tristis* (Figs 13–16), which has a yellow costal patch extending to the wing apex, *G. stueningi* sp. nov. shows white spots along the upper half of the postmedial line. Although molecular evidence supports the closest relationship of this new species with *G. flavescens* (Figs 11, 12), *G. stueningi* sp. nov. can be distinguished by the absence of secondary sexual characteristics.

#### Distribution.

China (Shaanxi, Gansu, Sichuan).

#### Etymology.

The species is named after Dr Dieter Stüning (Bonn, Germany), former curator of Lepidoptera at the Zoological Research Museum Alexander Koenig, Bonn, and a well-known specialist, for his significant contributions to Geometridae taxonomy.

#### Molecular data.

*Gandaritis stueningi* is clearly separated from closely related species based on COI barcode sequences (Fig. 1). The genetic distance between *G. stueningi* and *G. flavescens* is 2.11% to 2.77%, and between *G. stueningi* and *G. flavomacularia* is 5.13% to 5.30%. The mean intraspecific distance of *G. stueningi* is 0.68% (range 0–1.07%; *N* = 4).

## Supplementary Material

XML Treatment for Gandaritis stueningi

## ﻿Additional information

### Use of AI

No use of AI was reported.

### Author ORCIDs

Boxin Wen https://orcid.org/0009-0008-6405-4733

Rui Cheng https://orcid.org/0000-0002-3044-4724

### Data availability

All of the data that support the findings of this study are available in the main text or Supplementary Information.

## ﻿Appendix 1

**Table A1.** Details of specimens used in the molecular analysis of the DNA barcode region.

Sample ID	Species	Date collected	Locality	Collectors	GenBank /BOLD accession numbers
IOZ LEP M 22875	*G. flavomacularia*	1.VIII.2016	Baoxing, Sichuan, China	Cui Le	PV918580
IOZ LEP M 22510	*G. flavomacularia*	15.VII.2016	Jiguanshan, Sichuan, China	ditto	PV918581
IOZ LEP M 22674	*G. stueningi*	23.VII.2016	Pingwu, Sichuan, China	ditto	PV918582
IOZ LEP M 22602	*G. stueningi*	ditto	ditto	ditto	PV918583
IOZ LEP M 15798	*G. stueningi*	8.VIII.2014	Tiantaishan, Shaanxi, China	Xue Dayong	PV918584
IOZ LEP M 15862	*G. stueningi*	ditto	ditto	Ban Xiaoshuang	PV918585
KLM Lep 02811	*G. pyraliata*	22.VI.2014	Kaernten, Austria	Wieser Ch.	OR369486
TLMF Lep 18953	*G. pyraliata*	28.VII.2013	Tyrol, Osttirol, Austria	Deutsch H.	OR369353
LSNOE Lep 00989	*G. pyraliata*	21.VIII.2010	Niederoeste-rreich, Austria	W. Stark	OR369308
TLMF Lep 23926	*G. pyraliata*	14.VI.2017	Burgenland, Austria	Huemer P.	OR369208
AYK-04-1280-07	*G. sinicaria*	2.VIII.2004	Taiwan, China	Akito Y. Kawahara	KF522414
AYK-04-5649	*G. fixseni*	1.VIII.2008	Shizuoka, Japan	ditto	KF522413
BC AxYi 0025	* flavescens *	25.VII.2011	Beijing, China	Zou Y	GWOTL785-13
MF052653	* flavescens *		Beijing, China	Hao MD	GBMNC53060-20

## Supplementary materials

Supplementary material 1 DNA sequence Data type txt https://binary.pensoft.net/file/1440693This dataset is made available under the Open Database License (http://opendatacommons.org/licenses/odbl/1.0/). The Open Database License (ODbL) is a license agreement intended to allow users to freely share, modify, and use this Dataset while maintaining this same freedom for others, provided that the original source and author(s) are credited.Boxin Wen, Rui Cheng
